# A new assay for measuring chromosome instability (CIN) and identification of drugs that elevate CIN in cancer cells

**DOI:** 10.1186/1471-2407-13-252

**Published:** 2013-05-22

**Authors:** Hee-Sheung Lee, Nicholas CO Lee, Brenda R Grimes, Alexander Samoshkin, Artem V Kononenko, Ruchi Bansal, Hiroshi Masumoto, William C Earnshaw, Natalay Kouprina, Vladimir Larionov

**Affiliations:** 1 Laboratory of Molecular Pharmacology, National Cancer Institute, Bethesda, MD 20892, USA; 2 Department of Medical and Molecular Genetics, Indiana University School of Medicine, Indiana University Melvin and Bren Simon Cancer Center, Indianapolis, IN 46202, USA; 3 Laboratory of Cell Engineering, Department of Human Genome Research, Kazusa DNA Research Institute, 2-6-7 Kazusa-Kamatari, Kisarazu, Chiba 292-0818, Japan; 4 Wellcome Trust Centre for Cell Biology, University of Edinburgh, Edinburgh EH9 3JR, Scotland

**Keywords:** Human artificial chromosome, HAC, Chromosome instability, CIN, Drug treatment

## Abstract

**Background:**

Aneuploidy is a feature of most cancer cells that is often accompanied by an elevated rate of chromosome mis-segregation termed chromosome instability (CIN). While CIN can act as a driver of cancer genome evolution and tumor progression, recent findings point to the existence of a threshold level beyond which CIN becomes a barrier to tumor growth and therefore can be exploited therapeutically. Drugs known to increase CIN beyond the therapeutic threshold are currently few in number, and the clinical promise of targeting the CIN phenotype warrants new screening efforts. However, none of the existing methods, including the *in vitro* micronuclei (MNi) assay, developed to quantify CIN, is entirely satisfactory.

**Methods:**

We have developed a new assay for measuring CIN. This quantitative assay for chromosome mis-segregation is based on the use of a non-essential human artificial chromosome (HAC) carrying a constitutively expressed *EGFP* transgene. Thus, cells that inherit the HAC display green fluorescence, while cells lacking the HAC do not. This allows the measurement of HAC loss rate by routine flow cytometry.

**Results:**

Using the HAC-based chromosome loss assay, we have analyzed several well-known anti-mitotic, spindle-targeting compounds, all of which have been reported to induce micronuclei formation and chromosome loss. For each drug, the rate of HAC loss was accurately measured by flow cytometry as a proportion of non-fluorescent cells in the cell population which was verified by FISH analysis. Based on our estimates, despite their similar cytotoxicity, the analyzed drugs affect the rates of HAC mis-segregation during mitotic divisions differently. The highest rate of HAC mis-segregation was observed for the microtubule-stabilizing drugs, taxol and peloruside A.

**Conclusion:**

Thus, this new and simple assay allows for a quick and efficient screen of hundreds of drugs to identify those affecting chromosome mis-segregation. It also allows ranking of compounds with the same or similar mechanism of action based on their effect on the rate of chromosome loss. The identification of new compounds that increase chromosome mis-segregation rates should expedite the development of new therapeutic strategies to target the CIN phenotype in cancer cells.

## Background

An abnormal chromosome number (aneuploidy) is a feature of most solid tumors and is often accompanied by an elevated rate of chromosome mis-segregation termed chromosome instability (CIN) [1]. The gain or loss of entire chromosomes leads to large-scale changes in gene copy number and expression levels. The molecular mechanisms underlying CIN include defects in chromosome cohesion, mitotic checkpoint function, centrosome copy number, kinetochore-microtubule attachment dynamics, and cell-cycle regulation. While CIN can act as a driver of cancer genome evolution and tumor progression, recent findings point to the existence of a threshold level beyond which CIN becomes a barrier to tumor growth, and therefore, it can be exploited therapeutically. Janssen and co-authors [2] have analyzed the consequences of gradual increases in chromosome segregation errors on the viability of tumor cells and normal human fibroblasts. Partial reduction of essential mitotic checkpoint components in tumor cell lines caused mild chromosome mis-segregation, but no lethality. These cells were, however, much more sensitive to low doses of taxol, which enhances the amount and severity of chromosome segregation errors. Sensitization to taxol was achieved by reducing the levels of Mps1 or BubR1, proteins with dual roles in checkpoint activation and chromosome alignment. Importantly, untransformed human fibroblasts with reduced Mps1 levels could not be sensitized to sub-lethal doses of taxol. Thus, targeting the mitotic checkpoint and chromosome alignment simultaneously may selectively kill tumor cells. In another study [3], a set of genes was identified that are repressed in response to taxol treatment and over-expressed in tumors exhibiting CIN. The silencing of these genes caused cancer cell death, suggesting that these genes might be involved in the survival of aneuploid cells. In diploid cells, but not in chromosomally unstable cells, taxol causes the repression of CIN-survival genes, followed by cell death. Taking into account the fact that aneuploidization *per se* seems to be a very inefficient path towards cancer and additional hits are necessary for the generation of a cancer cell ([4] and references therein), these and other studies [5,6] indicate that increased destabilization of chromosomes might push genetically unstable cancer cells towards death, whereas more stable normal cells would be able to tolerate such insults.

Elevation of CIN as an approach to cancer therapy is attracting considerable attention [2-5]. However, none of the methods used to study CIN and its induction by environmental agents is entirely satisfactory. Karyotype analysis is bedeviled by the karyotypic variation already often present in cancer cell lines. Micronucleus assays (MNi) are widely used to detect broken or lagging chromosomes, but fail to detect non-balanced chromosome segregation [7].

In this study, we developed a new assay for measuring CIN. This quantitative assay for chromosome mis-segregation is based on the use of the human artificial chromosome (HAC) constructed in our lab earlier as a gene therapy tool for the efficient and regulated expression of genes of interest [8-10]. The HAC contains centromeric repeats that form a functional centromere/kinetochore, allowing its stable inheritance as a nonessential chromosome, albeit with a loss rate roughly 10× that of the native chromosomes [11,12]. To adopt this HAC for CIN studies, an *EGFP* transgene was inserted into the HAC. This allowed the measurement of the HAC loss rate by routine flow cytometry. Thus, the HAC offers a sensitized and simple system to measure CIN, particularly after drug treatment. In this study, the HAC-based CIN assay has been verified using a set of well-known aneugens and clastogens. This new assay has the potential to be developed for high-through put screening methods to identify new compounds that elevate chromosome mis-segregation and drive lethal aneuploidy. New and potentially less toxic agents that selectively elevate CIN in cancer cells to promote cancer cell death identified with this new screening tool could lay the foundation for new treatment strategies for cancer.

## Methods

### Cell lines

Human fibrosarcoma HT1080 cells were cultured in Dulbecco’s modified Eagle’s medium (DMEM) (Invitrogen) supplemented with 10% (v/v) tet system-approved fetal bovine serum (Clontech Laboratories, Inc.) at 37°C in 5% CO_2_. Hypoxanthine phosphoribosyltransferase (HPRT)-deficient Chinese hamster ovary (CHO) cells (JCRB0218) carrying the alphoid^tetO^-HAC were maintained in Ham's F-12 nutrient mixture (Invitrogen) plus 10% FBS with 8 μg/ml of BS (Funakoshi). After loading of the *EGFP* transgene cassette into the alphoid^tetO^-HAC, the CHO cells were cultured in 1× HAT supplemented medium.

### Loading of the *EGFP* transgene cassette into the loxP site of alphoid^tetO^-HAC in CHO cells

A total of 3 to 5 μg of a *EGFP* transgene plasmid (or X3.1-I-EGFP-I described previously [13]) and 1 to 2 μg of the Cre expression pCpG-iCre vector DNA were co-transformed into HPRT-deficient CHO cells containing the alphoid^tetO^-HAC by lipofection with FuGENERHD transfection reagent (Roche) or Lipofectamine 2000 (Invitrogen). HPRT-positive colonies were selected after 2 to 3 weeks growth in HAT medium. For each experiment, from 5 to 7 clones were usually selected. Correct loading of the *EGFP* transgene cassette into the HAC was confirmed by genomic PCR with a specific pair of primers that diagnose reconstitution of the *HPRT* gene [9].

### Microcell-mediated chromosome transfer

The alphoid^tetO^-HAC containing the *EGFP* transgene cassette (EGFP-HAC) was transferred from CHO cells to HT1080 cells using a standard microcell-mediated chromosome transfer (MMCT) protocol [13,14]. Blasticidin (BS) was used to select resistant colonies containing the HAC. Typically, three to ten BS^R^ colonies were obtained in one MMCT experiment. BS^R^ colonies were analyzed by FISH for the presence of the autonomous form of the HAC. The co-transfer of CHO chromosomes was examined using a sensitive PCR test for rodent-specific *SINE* elements [9].

### Flow cytometry

Analysis of EGFP expression was performed on a FACS Calibur instrument (BD Biosciences) using CellQuest acquisition software and analyzed statistically with FlowJo software [15,16]. The cells were harvested by trypsin-treatment. Intensities of fluorescence were determined by flow cytometry. A minimum of 4 x 10^4^ cells was analyzed for each cell sample.

### Drug treatment

Nine different drugs were used in our experiments (Table 1). Our experiment protocol was as follows. HT1080 cells containing the EGFP-HAC were maintained on blasticidin selection to select for the presence of the HAC. Approximately 1 × 10^5^ cells were cultured either in the presence or absence of blasticidin selection in parallel with a third culture that was exposed to the agent under examination to test its effect on EGFP-HAC segregation. The drug concentration applied was adjusted to the IC50 level for each compound which was determined using a proliferation assay described below. Concentrations of drugs and lengths of treatment are presented in Table 1. Subsequently, the drug was removed by performing three consecutive medium washes and the cells were subsequently grown without blasticidin selection for 1–14 days. At the end of the experiment, cells were collected and analyzed by flow cytometry to detect the proportion of cells that retain EGFP fluorescence. This served as a measure of EGFP-HAC stability following drug treatment. For taxol and peloruside A, nine independent measuring of EGFP-HAC loss were carried out. The results were reproducible and the std were small (peloruside A: SD±0.9%, taxol: SD±1.1%). Therefore, for other drugs, experiments were carried out in triplicate.

**Table 1:** Drugs used in this study

**Drug**	**Target**	**Concentration**/ **time treatment**	**Fold increase of HAC loss per cell division**
**Microtubule**-**stabilizing drugs**			
Taxol	Beta-tubulin	10 nM-overnight	x 47
Ixabepilone	Beta-tubulin	100 nM-overnight	x 31
Docetaxel	Beta-tubulin	10 nM-2 hrs	x 10
Peloruside A	Beta-tubulin	100 nM-overnight	x 32
**Microtubule**-**depolymerizing drugs**			
Nocodazole	Beta-tubulin	1 μM-overnight	x 8
**Other drugs**			
SAHA	HDAC	5 μM-overnight	x 1
VP16 (etoposide)	TOP2	8 μM-2hrs	x 7
Reversine	Aurora B, MPS1	1 μM-3 days	x 14
ZM-447439	Aurora B	1 μM-3 days	x 29

### Calculation of the rate of HAC loss after drug treatment

**Figure 1:**
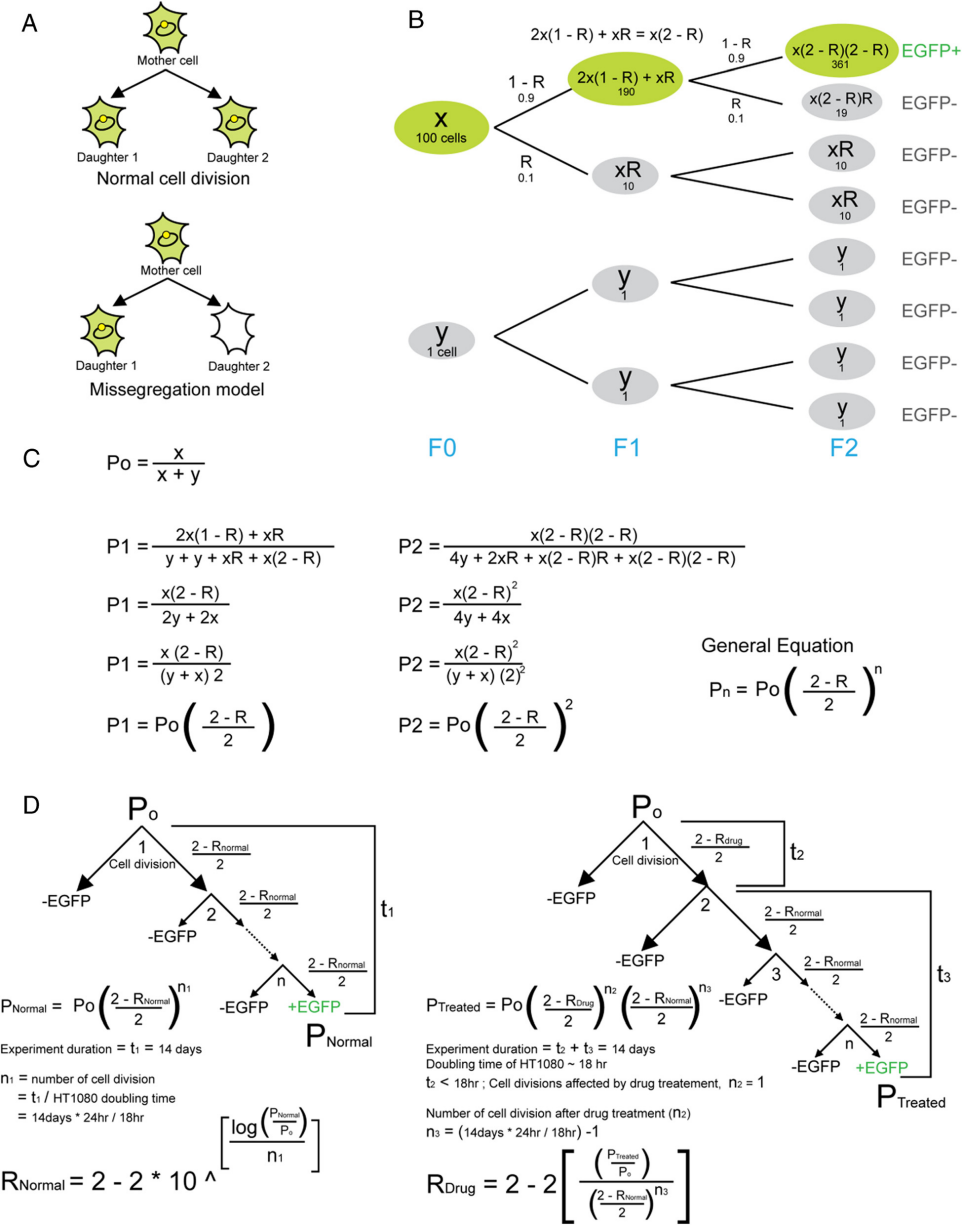
**Calculation of the rate of HAC miss**-**segregation induced by drug treatment.** Justification of the algorithm describing the dynamics of the accumulation of HAC-less cells caused by a single dose of chromosome-destabilizing compounds. This mathematical model assumes: 1) the drug kills cells non-selectively; 2) the drug’s effect on HAC mis-segregation is not persistent and limited to the cell cycle when it is present; 3) spontaneous HAC loss after drug exposure does not change; 4) the HAC does not confer a selective advantage or disadvantage; 5) the cells are growing synchronously and 6) there is one HAC per cell. Assumptions 2) and 3) have been confirmed experimentally (see Additional file 1). (**A**) Our model assumes that when mis-segregation occurs during mitosis, one daughter cell will inherit a HAC while the other daughter cell does not. (**B**) Illustrated model of HAC lost in a population of cells. (**C**) Derivation of the general equation for HAC miss-segregation rate. x is the number of cells which are EGFP(+); y is the number of cell which are GFP(−); R is the probability of HAC miss-segregation; n is the number of cell divisions; P_0_ is the proportion of EGFP(+) cell at generation F_0_; P_1_ is the proportion of EGFP(+) cell at generation F_1_. (**D**) Calculation of rates (see Methods for more details).

The formula, *P*_*n*_ = *P*_0_(1 − *R*)^*n*^[17], routinely used to calculate the rate (R) of spontaneous HAC (or chromosome) loss, cannot be applied when cells are treated by a single dose of drug. So in our study, we first determined the normal rate of spontaneous HAC miss-segregation (R_Normal_) in the host cell line HT1080 using the formula, $P_{\mathrm{normal}}=P_{0}{\left(\frac{2-R_{\mathrm{Normal}}}{2}\right)}^{n_{1}}$ (Figure 1); where P_0_ is the percentage of EGFP(+) cells at the start of the experiment as determined by FACS. These cells were cultured under HAC selection conditions using blaticidin. P_Normal_ is the percentage EGFP(+) cells after culturing without HAC selection (no blasticidin) for a duration of t_1._ In this study t_1_ was 14 days. n_1_ is the number of cell doublings that occurs during culturing without blasticidin selection. The doubling time of HT1080 under normal growth conditions is approximately 18 hours. The number of cell divisions (n) is calculated by (t / host cell doubling time).

Once (R_Normal_) was obtained, the rate of HAC loss induced by drug treatment (R_Drug_) is then determined using the formula, $P_{\mathrm{Treated}}=P_{0}{\left(\frac{2-R_{\mathrm{Drug}}}{2}\right)}^{n_{2}}{\left(\frac{2-R_{\mathrm{Normal}}}{2}\right)}^{n_{3}}$. Justification of this algorithm is presented in Figure 1. As before, P_0_ represents the percentage of EGFP(+) cells at the start of the experiment, cultured under HAC selection condition. P_Treated_ is the percentage of EGFP(+) cells at the end of a drug treatment experiment with a duration of (t_2_ + t_3_), where t_2_ is the duration of drug treatment and t_3_ is the duration of culturing after the drug is removed. (t_2_ + t_3_) was 14 days in this study. n_2_ is the number of cell doublings that occurs during drug treatment, while n_3_ is the number of cell doublings that occurs during the culturing without selection after drug treatment.

In the present study, the duration of most drug treatments were less than the duration of a single cell cycle of HT1080 (t_2_ <18 hr). We made the assumption that any significant increase in HAC loss occurs only during the first mitotic division after washing off a drug (n_2_ = 1). Thus n_3_ = (14 d / 18 hr - 1). The exceptions, reversine and ZM-447439 both severely inhibits cell growth, thus despite the 3 day drug treatment only one cell division is assumed to have occurred. We also assumed that HAC stability in subsequent cell divisions is no different from that of untreated cells.

The algorithm we used is valid between the ranges of 0 to 1. R values large than 1 indicate that the assumptions made in this model are incorrect. The assumption of synchronous growth in the model means that the estimated mis-segregation rate is lower than real values. As the spontaneous rate of HAC mis-segregation (R_Normal_) was found to be low, this algorithm is relatively insensitive to the number of cell divisions that occurs post drug treatment. It is worth noting that the frequency of HAC loss in the clones carrying two copies of the HAC is indistinguishable from those containing a single copy of the HAC (data not shown). Therefore, the model is applicable when a cell inherits two copies of the HAC due to non-disjunction.

### Cell viability test

MTS tetrazolium cell viability assays were done according to the manufacturer’s instructions (CellTiter 96 AQueous Assay reagent; Promega). Briefly, the CellTiter 96 AQueous One Solution Reagent was added to each well and incubated at 37°C for 3 h. Cell proliferation was determined by measuring the absorbance at 490 nm using a microtiter plate reader (Molecular Devices, Sunnyvale, CA). The half-maximal inhibitory concentration (IC50) was obtained from the MTS viability curves using GraphPad Prism 5. Experiments were carried out in triplicate.

### FISH analysis with PNA probes

The presence of the HAC in an autonomous form was confirmed by FISH analysis as previously described [8,9]. HT1080 cells containing the HAC were grown in DMEM medium to 70-80% confluence. Metaphase cells were obtained by adding colcemid (Gibco) to a final concentration of 0.05 μg/ml and incubating overnight. Media was aspirated, and the plate washed with 1x PBS. Cells were removed from the plate by 0.25% Typsin, washed off with DMEM, pelleted and resuspended in 10 ml of 50 mM KCl hypotonic solution for 30 min at 37°C. Cells were fixed by three washes of fixative solution (75% acetic acid, 25% methanol). Between each wash, cells were pelleted by centrifugation at 900 rpm for 4 min. Metaphase cells were evenly spread on a microscope slide and the fixative solution evaporated over boiling water. Dry slides were rehydrated with 1× PBS for 15 min, and fixed in 4% formaldehyde-1× PBS for 2 min, followed by three 5 min 1× PBS washes and ethanol series dehydration. PNA (peptide nucleic acid) labeled probes used were telomere (CCCTAA)_3_-Cy3) (PerSeptive Biosystems, Inc.) and tetO-alphoid array (FITC-OO-ACCACTCCCTATCAG) (Panagene, South Korea). Ten nanomol of each PNA probe was mixed with hybridization buffer and applied to the slide, followed by denaturation at 80°C for 3 min. Slides were hybridized for 2 hours at room temperature in the dark. Slides were washed twice in 70% formamide, 10 mM Tris pH 7.2, 0.1% BSA and followed by three washes with 1xTBS, 0.08% Tween-20. Slides were dehydrated gradually with a series of 70%, 90% and 100% ethanol washes and mounted (Vectorshield with DAPI). Images were captured using a Zeiss Microscope (Axiophot) equipped with a cooled-charge-coupled device (CCD) camera (Cool SNAP HQ, Photometric) and analyzed by IP lab software (Signal Analytics). The PNA-DNA hybrid probes demonstrated a high hybridization efficiency, staining intensity and adopt a stable duplex form with complementary nucleic acid.

### FISH analysis with the BAC probe

HT1080 cells were processed for fluorescence in situ hybridization (FISH) after drug treatment followed by the 14 day washout. The probe used for FISH was BAC32-2-mer(tetO) DNA containing 40 kb of alphoid-tetO array cloned into a BAC vector as described previously [8]. Specifically, a BAC32-2-mer(tetO) clone contains an amplified synthetic alphoid DNA dimer. One monomer of this dimer is an alphoid DNA consensus sequence carrying the tetO sequence; another monomer is alphoid DNA from chromosome 17. This probe is specific to the HAC but also gives a low signal with centromeric regions of several endogenous chromosomes. BAC DNA was digoxigenin-labeled using a nick-translation kit with digoxigenin-11dUTP or biotin16-dUTP (Roche Diagnostics). Images were captured as before. The probe was denatured for 5 min at 95°C and added to the slides, which were incubated at 72°C for 2 min before overnight incubation at 39°C. After washes with 0.1 × SSC at 65°C followed by a wash with 4 × SSC + 0.1% Tween 20 at room temperature, standard procedures were used to detect biotinylated probes. Slides were mounted with VectaShield and screened for the presence or absence of the HAC. Between 70–250 metaphases were analyzed for each experiment (one drug treatment).

### Genomic DNA preparation and PCR analysis

Genomic DNA for PCR analysis was prepared using a QIAmp DNA Mini Kit (QIAGEN Inc., Valencia, CA, USA). Reconstitution of the *HPRT* gene after Cre/lox-mediated recombination was determined by specific primers, Lox137-R 5′-agccttctgtacacatttcttctc-3′ and Rev #65′-gctctactaagcagatggccacagaactag-3′. Cross contamination by hamster chromosomes was determined by specific primers detecting hamster *SINE*s: Cons B2 5′-ccatctgtaatgagatctgatgc-3′, Ham B2-F 5′-gctcagaggttaagagcactgac-3′ and Ham B2-R 5′-tgcttccatgtatatctgcacac-3′. PCR products for sequencing were separated by agarose gel electrophoresis and gel extracted.

### Micronucleus formation assay (MNi)

Duplicate cultures of cells were exposed to different compounds or DMSO control. Micronuclei formation was assessed by phase contrast microscopy in Table 2 after 20 hours. In the time course assay (Additional file 1), cells were harvested after 20 h treatment and grown in compound-free medium for 24 h, 5 days and 10 days then placed on chamber slides. Dihydrocytochalasin B was added for 90 min to permit formation of cytokinesis blocked cells. Cells were washed with PBS and incubated with 0.075 M KCl then fixed in ice cold 3:1 v/v methanol: acetic acid and dried under vacuum. The frequency of micronucleated cells was assessed as described in [18] using DAPI staining and fluorescence microscopy.

**Table 2:** Micronuclei (MNi) formation in the HAC-containing HT1080 cells treated by different drugs

**Drug**	**Fraction of cells with MNi**		Mean ± SD (%)**
	**Exp. 1**	**Exp. 2**	
Vehicle*	3%	1%	2 ± 1
10 nM Taxol	43%	37%	40 ± 3
100 nM Peloruside A	74%	63%	69 ± 5
1 μM ZM447439	56%	57%	57 ± 1
100 nM Nocodazole	50%	56%	53 ± 3

*Vehicle: 0.01% DMSO (this was the highest amount of DMSO used).

** Number of MN is given as means ± SD in two independent experiments.

## Results

### Experimental design for identification of drugs that elevate CIN in cancer cells

**Figure 2:**
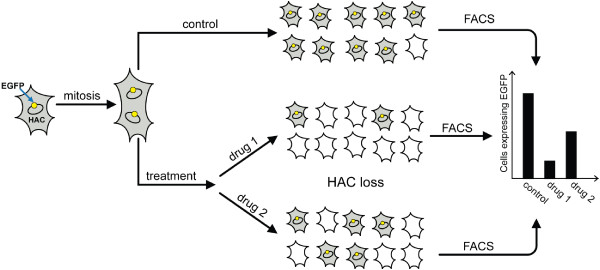
**Scheme of an assay for measuring chromosome instability (CIN) based on the use of HAC containing the*EGFP*transgene.** Cells that inherit the HAC display green fluorescence, while cells that lack it do not. It is expected that the control untreated cells should show uniform green fluorescence, while those that have lost HAC after drug treatment should be highly variable in fluorescence. Therefore, the actual number of cells with the EGFP-HAC can be measured by FACS. Thus, the compounds, which increase HAC loss and therefore increase spontaneous chromosome mis-segregation rates, may be identified.

Figure 2 shows a general scheme of the new assay developed for measuring chromosome instability (CIN) based on the use of a human artificial chromosome (HAC) carrying the *EGFP* (enhanced green fluorescence protein) transgene. Due to the presence of a functional kinetochore, the EGFP-HAC is maintained as a non-essential 47th chromosome that replicates and segregates like a normal chromosome in human cells. Thus, the cells that inherit the HAC display green fluorescence, while cells that lack it do not. Normally, after growing in non-selective medium (i.e. in the absence of selection for the HAC), the majority of cells contain one copy of the HAC. After drug treatment, there are two possibilities: either no change in the EGFP level (no response to the drug) or an increased percentage of cells without HAC due to HAC segregation or replication errors (response to the drug treatment). It is expected that the control untreated cells should show uniform green fluorescence, while those that have lost HAC after drug treatment will exhibit reduced fluorescence that can be detected by flow cytometry.

### Construction of the HAC carrying a single copy of the *EGFP* transgene and its transfer to human cells

**Figure 3:**
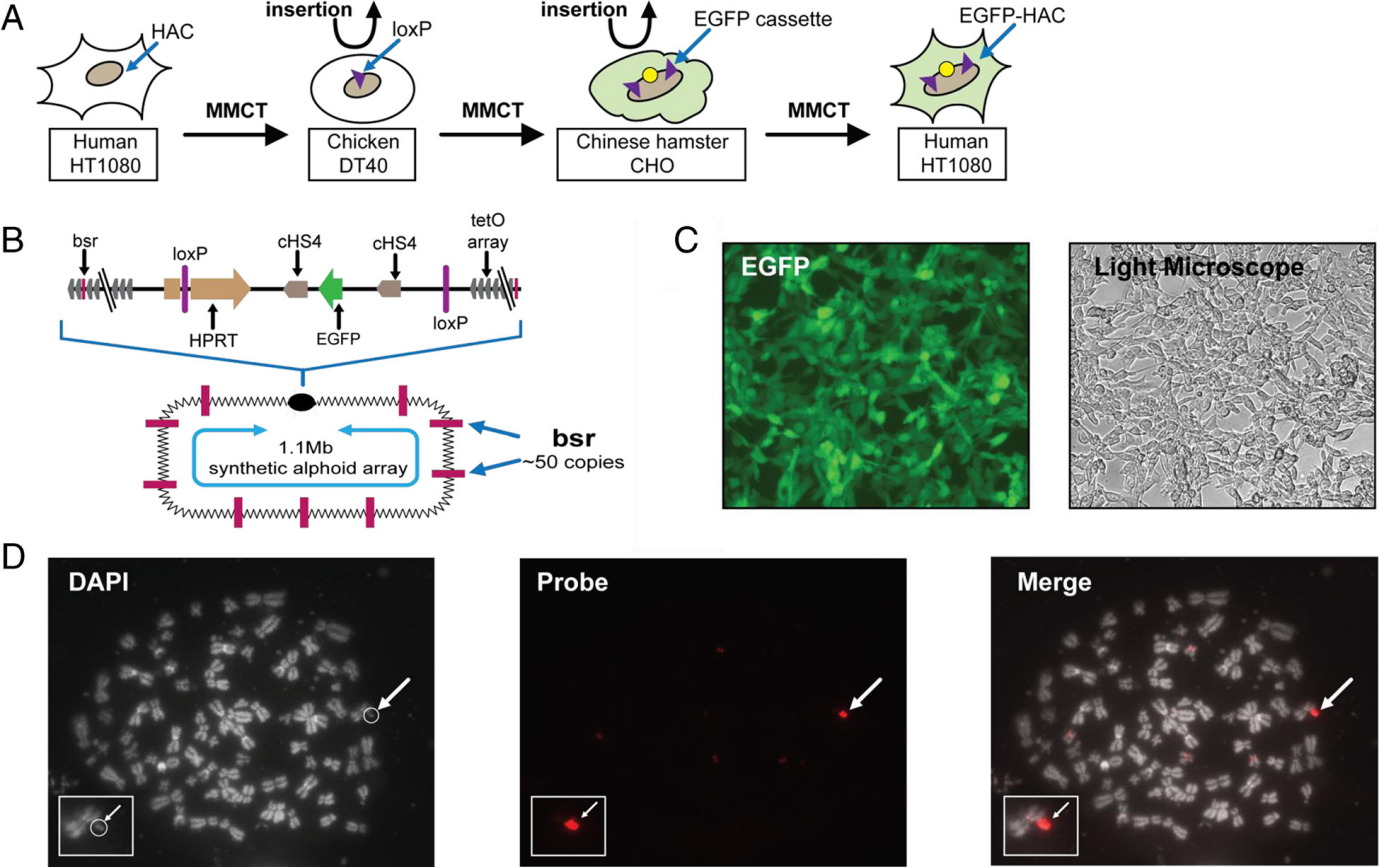
**Schematic diagram of construction of the alphoid**^**tetO**^-**HAC containing the*EGFP*transgene to measure chromosome instability.** (**A**) Three steps of MMCT to transfer the HAC. The original alphoid^tetO^-HAC was generated in human fibrocarcoma HT1080 cells [8]. The alphoid^tetO^-HAC was transferred to homologous recombination proficient chicken DT40 cells via MMCT. In chicken DT40 cells, a loxP gene loading site was generated in the HAC [13]. The modified alphoid^tetO^-HAC was transferred to HPRT-deficient hamster CHO cells via MMCT. (**B**) Loading of the *EGFP*-containing cassette into the HAC was carried out in hamster CHO cells. Insertion of the cassette into the loxP site of the HAC by Cre/lox-mediated recombination is accompanied by reconstitution of the *HPRT* gene allowing the cells selection on the HAT medium. This modified HAC was transferred back to human HT1080 cells via third round of MMCT. (**C**) Fluorescence images of cells carrying the HAC with the *EGFP* cassette are shown. (**D**) FISH analysis of the HAC-containing HT1080 clone. The HAC was visualized using BAC32-2-mer(tetO) DNA containing 40 kb of alphoid-tetO array cloned into a BAC vector as described previously [8] (red). Chromosomal DNA was counterstained with DAPI. The HAC is indicated by arrowhead.

The *EGFP* transgene cassette (X3.1-I-EGFP-I) was described previously [13]. To adapt the alphoid^tetO^-HAC [8] for CIN studies, the *EGFP* transgene cassette was inserted into a single loxP loading site of the HAC [13] in hamster HPRT-deficient Chinese Hamster Ovary (CHO) cells (Figure 3A, B, 2B). In this cassette, *EGFP* is flanked by the *cHS4* insulator sequences to protect the transgene from epigenetic silencing. Targeting of the EGFP-cHS4 cassette into the loxP site was accompanied by reconstitution of the *HPRT* gene, allowing cell selection on HAT medium. Therefore, recombinant clones were selected by growth in HAT medium after two-three weeks. PCR analysis with specific primers (see Methods) confirmed that the *HPRT* gene was indeed reconstituted in all five drug-resistant clones analyzed (data not shown). The efficiency of cassette targeting into the HAC was <1-3 × 10^−4^. All of the HPRT^+^ transfectants expressed the *EGFP* transgene, which was detected by fluorescence microscopy. FISH analysis of CHO metaphase spreads revealed the HAC in an autonomous form in four clones (data not shown). One clone containing the HAC was chosen for further experiments.

The HAC containing the *EGFP* transgene was transferred from CHO to human HT1080 fibrosarcoma cells via microcell-mediated chromosome transfer (MMCT). Recipient cells were selected using the *BS* resistance gene on alphoid^tetO^-HAC [8]. Ten BS-resistant clones that expressed the *EGFP* transgene were isolated from one MMCT experiment and analyzed using PCR with hamster-specific primers (see Methods) to rule out co-transfer of hamster chromosomes after MMCT. Fluorescence images of HT1080 cells carrying the HAC with the *EGFP* cassette are shown (Figure 3C). FISH analysis showed that the HAC was maintained autonomously without any detectable integration into the host genome in three out of five randomly selected clones. One clone (clone 12) containing an autonomously propagated EGFP-HAC was chosen for further analysis (Figure 3D). Based on the results from FISH analysis, the rate of spontaneous HAC loss was 7 x 10^-3^ per cell division. The rate of HAC loss measured by the accumulation of non-fluorescent cells during growth in the absence of selection for the HAC by FACS was very similar, 13 × 10^-3^ (Table 3), suggesting that non-fluorescent cells arise primarily through loss of the EGFP-marked HAC. Notably, mitotic stability of the EGFP-HAC is approximately 10-fold less than stability of natural chromosomes in HT1080 cells (~1 × 10^-3^) [11,12], making the system more sensitive for the detection of mis-segregation events. It is worth noting that in this clone, EGFP expression was stable for at least twelve months under selective conditions (data not shown). Based on these results, we conclude that the *EGPF* transgene on the HAC is stably expressed and that cells offer a sensitized system for analyzing chromosome loss.

**Table 3:** Comparison between FISH and FACS data to evaluate HAC induced by drug treatment

**Drug**	**FISH**	**FACS**	**Rate of HAC loss**
	**% Cells with HAC**	**% Cells with EGFP**	per cell division**
No drug	86 (100)*	85.1 ± 0.9	13 × 10^-3^
Taxol	59 (139)	59.5 ± 0.9	597 × 10^-3^
Ixabepilone	71 (116)	68.0 ± 0.1	396 × 10^-3^
Docetaxel	74 (101)	79.1 ± 1.8	133 × 10^-3^
Peloruside A	68 (71)	67.4 ± 0.9	411 × 10^-3^
Nocodazole	79 (77)	80.3 ± 0.9	107 × 10^-3^
SAHA	79 (160)	84.3 ± 1.2	12 × 10^-3^
VP16 (Etoposide)	86 (156)	80.7 ± 0.9	96 × 10^-3^
Reversine***	77 (71)	79.0 ± 0.5	176 × 10^-3^
ZM-447439***	75 (75)	70.4 ± 0.5	374 × 10^-3^

* In parentheses, the number of metaphases screened for the presence or absence of the HAC.

** Rates of the HAC loss after drug treatment were calculated from FACS data using the formula $P_{\mathrm{Treated}}=P_{0}{\left(\frac{2-R_{\mathrm{Drug}}}{2}\right)}^{n_{2}}{\left(\frac{2-R_{\mathrm{Normal}}}{2}\right)}^{n_{3}}$.

*** A real rate of HAC loss may be a little bit lower after treatment by inhibitors of Aurora B and MPS1 because reversine and ZM-447439 do not completely block cell divisions.

### Effect of aneugens and clastogens on the rate of HAC mis-segregation during mitotic divisions

We next investigated whether the EGFP/HAC-based assay could be used to detect compounds that cause chromosome loss and mis-segregation. HT1080 cells with an autonomously propagated EGFP-HAC were treated with eight known aneugens: taxol (pacilitaxel), docetaxel, peloruside A, ixabepilone, nocodazole, ZM447439, reversine, and SAHA (Table 1). Taxol, docetaxel, peloruside A, ixabepilone, and nocodazole are microtubule-targeting drugs [19]; ZM447439 and reversine are inhibitors of Aurora B and MPS1, respectively, and function in the spindle assembly checkpoint [20,21]. SAHA is an inhibitor of histone deacetylases (HDAC) [22]. Cells were also treated with the well-known clastogenic DNA topoisomerase II inhibitor VP16 (etoposide) [23].

**Figure 4:**
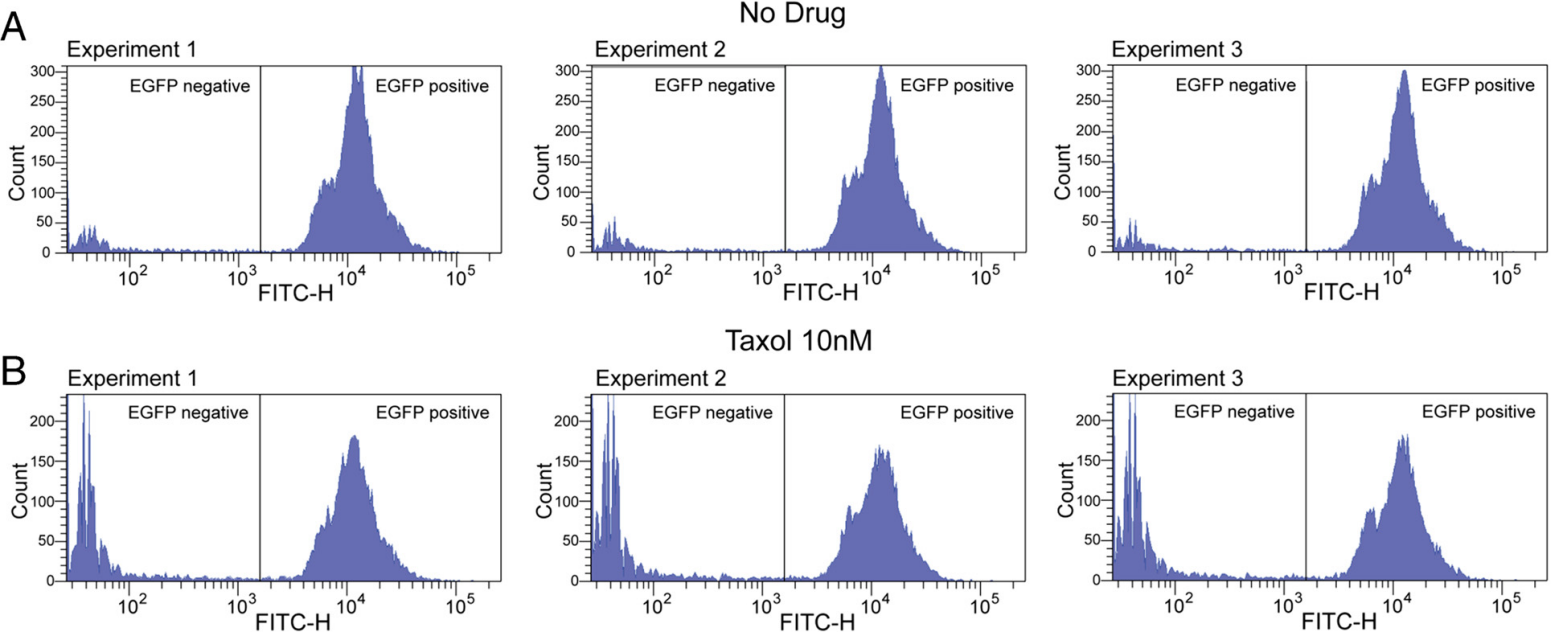
**Two flow cytometry histograms illustrating mitotic stability of the EGFP**-**HAC in HT1080 cells (A) before and (B) after treatment by taxol.** The x-axis represents the intensity of the fluorescence, the y-axis the number of cells. The bar stands for the amount of positive cells. The results of triplicate experiments are shown.

For each compound, a cell cytotoxicity assay was carried out to determine IC50 values, i.e., the conditions under which the viability of cells would be around 50%. We chose this parameter in order to normalize the results at the same percentage of viable cells. The evaluation of chromosome instability at the IC50 values has been used in many studies involving micronucleus scoring [24,25]. Time treatments and drug concentrations corresponding to IC50 are summarized in Table 1. For all compounds, the IC50 values were in the range clinically relevant concentrations, where applicable. After treatment, the cells were grown for two weeks in the absence of selection. Samples were analyzed every few days, and the proportion of non-fluorescent cells was determined. Non-fluorescent cells could be detected after a few days, and treated and untreated cell populations were clearly distinguishable after 5–7 days. The delay between HAC loss and the appearance of non-fluorescent cells is due to the persistence of the EGFP protein, which has a quite high half-life. Based on our analysis, sampling time has a broad interval (5–14 days) without a significant effect on the calculated rate of HAC loss (see Additional file 1). In most experiments, the cells were analyzed 14 days after drug washout. Figure 4 shows representative flow cytometry histograms illustrating loss of fluorescence for the cells treated by a single doze of taxol. It is important that the measuring is highly reproducible: The raw FACS data of three independent populations for drug treatments have standard deviations less than 1%.

**Figure 5:**
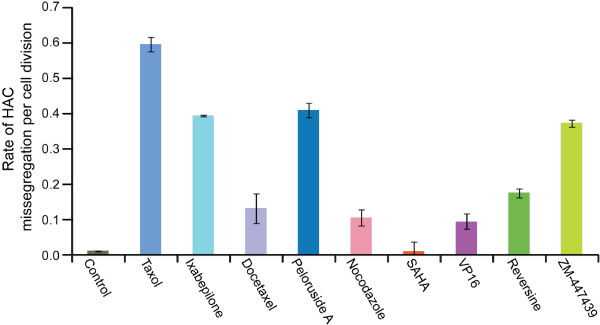
**Mitotic stability of the EGFP**-**HAC in HT1080 human cells treated by nine different drugs.** A rate of HAC loss per generation was calculated as described in Methods and Figure 1. Blue bars correspond to the frequency of HAC loss when the cells were treated by taxol and its derivatives. The control corresponds to a frequency of spontaneous loss of the EGFP-HAC in human HT1080 cells.

Figure 5 summarizes the estimated rates of HAC loss in response to different drugs. The rate of HAC loss (R_Drug_) induced by drug treatment was calculated from the proportion of non-fluorescent cells in the population (Table 3) using the formula $P_{0}{\left(\frac{2-R_{\mathrm{Drug}}}{2}\right)}^{n_{2}}{\left(\frac{2-R_{\mathrm{Normal}}}{2}\right)}^{n_{3}}$ (see more details in Methods and Figure 1 legend).

As seen, a single dose of taxol greatly increases (~50 times) the rate of HAC loss. Taxol (pacilitaxel) was isolated ~ 40 years ago and is currently administered in a large variety of indications, including solid tumors and haematological malignancies [19] and references therein]. While its mechanism of action remains controversial, accumulating data suggest that at clinically relevant concentrations, taxol-mediated cell death involves elevation of chromosome mis-segregation that is incompatible with cancer cell survival [26] and references therein]. HAC loss was also significantly increased by peloruside A, ixabepilone, which also binds to the microtubule as taxol, and by the Aurora B inhibitor ZM-447439. For the other analyzed compounds except SAHA, the rates of HAC loss were also increased compared to controls, but much lower compared to those obtained after taxol or peloruside A treatment. The lowest increase of HAC loss was obtained after treatment by the inhibitor of histone deacetylases, SAHA.

In separate experiments, the relationship of HAC loss to chemical dose was investigated. Treatment of cells with higher doses of the compounds kills more cells but does not increase the rate of HAC loss. This may be explained if cells arrest, then apoptosis in response to damage of the spindle or if the daughter cells are not viable. We also performed the experiments with lower doses of drugs, i.e. with concentrations ½, ¼, ^1^/_8_ and ^1^/_16_ of the concentration corresponding to IC50. For some drugs, the frequency of HAC loss was dose independent while for others displayed a linear dose-dependence. For example, HAC loss was decreased in an almost linear fashion after treatment by VP16 (which is inhibitor of TOP2) with lower doses. In contrast, our analysis revealed a non-linear dose-dependent decrease in HAC loss frequencies for nocodazole and taxol, which were previously reported as compounds that interact with the mitotic spindle (thresholded concentration-effect response) [27].

FISH analysis was used to confirm that the appearance of non-fluorescent cells detected by flow cytometry corresponds to HAC loss events and to exclude possible alternative explanations such as mutations in the *EGFP* gene or its epigenetic silencing. Quantitative analysis of metaphase chromosome spreads using a HAC-specific probe (see Methods) correlated with the data on HAC loss determined by FACS (Table 3). Therefore, we conclude that the appearance of non-fluorescent cells is caused by HAC loss.

An unexpected result was the different effects of microtubule-binding drugs on HAC stability. Ixabepilone, docetaxel, and peloruside A are microtubule-stabilizing agents similar to taxol [19]. However, each drug exhibited a different effect on HAC mitotic stability, suggesting a different cell response to these compounds. Interestingly, under these conditions, all of the analyzed drugs induced micronuclei formation in the HAC-containing HT1080 cells (Table 2) similar to that reported for other cell lines [27]. However, there was no detectable correlation between frequencies of MNi formation (that varied between 37% and 63%) and rates of HAC loss determined by FISH and FACS (Table 3). Thus, aneugens with a similar mechanism of action and cytotoxicity may differ from each other by their effect on the mitotic stability of the non-essential human artificial chromosome. It is unlikely that karyotyping or another variant of MNi tests can detect such differences between the compounds. Based on these results, we conclude that a newly developed EGFP/HAC-based assay for measuring CIN is a more sensitized system than other previously described methods to detect chromosome mis-segregation.

## Discussion

Targeting of CIN in cancer therapy requires measurement of the accuracy of chromosome transmission. At present, a variety of methods is used to study chromosome instability (CIN) and its induction by environmental agents [7,28] and references therein]. Because test systems screening for numerical chromosomal effects rely on labor-intensive microscopic assessment, the micronucleus (MNi) formation test is the most widely used method for large-scale detection of broken or lagging chromosomes [7]. However, because the origins and fates of MNi have not been completely elucidated [29,30], intra- and inter-laboratory variability in scoring is still common [31], and complicates the development of a standard protocol for quantitative measurement of chromosome loss rates based on the appearance of MNi. It is also noteworthy that the MNi assay does not measure the fraction of drug-arrested cells that undergo mitosis and form viable aneuploid cells.

This work describes a new assay for measuring CIN in response to drug treatment that overcomes the limitations of current approaches. The assay is based on quantitative measurements of mitotic loss rates of a nonessential human artificial chromosome (HAC) carrying a transgene encoding the green fluorescent protein (EGFP). Thus, cells that inherit the HAC fluoresce green, while cells that lack it do not. The proportion of cells that lose the HAC in response to drugs is measured using FACS, which allows the objective and accurate assessment of chromosome loss in a large number of cells (e.g., 10^6^) within minutes. Because only viable cells are studied, the long-term effects of aneugen exposure on chromosome loss are readily measured. It is worth noting that a similar approach but involving natural chromosome was previously attempted [32], but proved not to be useful for CIN studies, as the rates of chromosome loss were too low to be measured accurately. In the present study, the use of a HAC reporter that is sensitized for chromosome loss circumvents this problem. The HAC contains a functional kinetochore and its behavior during mitotic divisions does not differ from that of normal chromosomes [8,10], suggesting that destabilization of natural chromosomes in response to drug treatment will be increased proportionally to that observed for the HAC.

As proof of principle, the EGFP/HAC-based assay was applied to analyze a set of well-known anti-mitotic, spindle-targeting compounds previously reported to induce micronucleus formation and chromosome loss. For each drug, the rate of HAC loss was measured by flow cytometry and confirmed by FISH analysis. The most interesting result obtained from these experiments is the observation that despite their similar cytotoxicity, the analyzed drugs affect the rates of HAC mis-segregation during mitotic divisions differently. Moreover, we found that aneugens with proposed similar mechanisms of action and cytotoxicity may greatly differ from each other by their effect on the mitotic stability of the non-essential human artificial chromosome. Thus, the assay described here is sensitive enough to discriminate between drugs with similar mechanisms of action in order to reveal those with the highest activity on chromosome stability. In future drug screening studies, taxol exhibiting the highest effect on HAC stability may be used as a positive control.

In this work to compare different compounds, the cells were analyzed 14 days after drug washout. At the same time, the sampling time for HT1080 cells has a broad interval and fluorescence of the treated cells can be measured even after 5–7 days without a significant effect on the calculated rate of HAC loss (see Additional file 1). For other cell lines, where the EGFP protein is more stable, the sampling time may be exceeded up to 10–14 days. However, the speed of the assay can be significantly increased using a modified EGFP with a reduced half-life (e.g., EGFP with a degron box sequence) that would convert an assay into high-throughput screening. The transfer of EGFP-HAC into other cell lines will make possible to assess effects of inhibitors in different genetic backgrounds such as cell lines harboring mutations in genes that regulate the spindle assembly checkpoint. It is worth noting that recent innovations have allowed the generation of HACs with the *EGFP* transgene *de novo* in a broad variety of human cell lines [33].

This new HAC-based flow cytometry assay may be also applied for identifying genes controlling chromosome segregation in human cells because it is more sensitive to perturbations of spindle assembly and cell cycle progression than assays based on the analysis of stability of natural chromosomes [34]. Earlier, the development of conceptually simple color colony assays in yeast provided a powerful genetic tool to assess the rates of chromosome mis-segregation and to identify mutants deficient in this process [35]. Similarly, in humans, the EGFP/HAC-based chromosome loss assay may be used to reveal new human genes involved in chromosome transmission.

## Conclusions

We have developed a new assay for measuring chromosome instability based on the use of a non-essential human artificial chromosome (HAC) carrying a constitutively expressed EGFP transgene. This simple assay allows for a quick and efficient screen of hundreds of drugs to identify those affecting chromosome mis-segregation. It also allows ranking of compounds with the same or similar mechanism of action based on their effect on the rate of chromosome loss.

## Competing interests

The authors declare they have no competing interests.

## Authors’ contributions

HSL, NCOL, BRG, AS, AVK performed the experiments. RB assisted with experiments. BRG, HM, WCE, NK, VL analyzed the data. VL designed the study. VL, NK, WCE, BRG contributed to writing the manuscript. All authors read and approved the final manuscript.

## Pre-publication history

The pre-publication history for this paper can be accessed here:

http://www.biomedcentral.com/1471-2407/13/252/prepub

## Supplementary Material

Additional file 1: Figure S1. (A) Micronuclei (MNi) formation (indicated by arrows) in the HAC-containing HT1080 cells treated by drugs. Scale bar = 10 μM. (B) The kinetics of MNi formation after drug treatment at different time points after washout. The experiment was performed for taxol (10 nM) and nocodazole (1 μM). (C) Rates of HAC loss calculated using FACS profiles of cells before, after drug treatment and at different time points after washout. The experiment was performed for taxol and ixabepilone. Click here for file

## References

[B1] Thompson SL Bakhoum SF Compton DA Mechanisms of chromosomal instability Curr Biol 2010 20 R285 R295 10.1016/j.cub.2010.01.034 20334839 PMC3781365

[B2] Janssen A Kops GJ Medema RH Elevating the frequency of chromosome mis-segregation as a strategy to kill tumor cells Proc Natl Acad Sci USA 2009 106 19108 19113 10.1073/pnas.0904343106 19855003 PMC2776415

[B3] Swanton C Nicke B Schuett M Eklund AC Ng C Li Q Hardcastle T Lee A Roy R East P Kschischo M Endesfelder D Wylie P Kim SN Chen JG Howell M Ried T Habermann JK Auer G Brenton JD Szallasi Z Downward J Chromosomal instability determines taxane response Proc Natl Acad Sci USA 2009 106 8671 8676 10.1073/pnas.0811835106 19458043 PMC2688979

[B4] Colombo R Moll J Targeting aneuploid cancer cells Expert Opin Ther Targets 2011 15 595 608 21314491 10.1517/14728222.2011.558007

[B5] Janssen A Kops GJ Medema RH Targeting the mitotic checkpoint to kill tumor cells Hormones & Cancer 2011 2 113 116 10.1007/s12672-010-0059-x 21475725 PMC3056011

[B6] Janssen A Van der Burg M Szuhai K Kops GJ Medema RH Chromosome segregation errors as a cause of DNA damage and structural chromosome aberrations Science 2011 333 1895 1898 10.1126/science.1210214 21960636

[B7] Kirsch-Volders M Elhajouji A Cundari E Van Hummelen P The in vitro micronucleus test: a multi-endpoint assay to detect simultaneously mitotic delay, apoptosis, chromosome breakage, chromosome loss and non-disjunction Mutat Res 1997 392 19 30 10.1016/S0165-1218(97)00042-6 9269328

[B8] Nakano M Cardinale S Noskov VN Gassmann R Vagnarelli P Kandels-Lewis S Larionov V Earnshaw WC Masumoto H Inactivation of a human kinetochore by specific targeting of chromatin modifiers Dev Cell 2008 14 507 522 10.1016/j.devcel.2008.02.001 18410728 PMC2311382

[B9] Kim JH Kononenko A Erliandri I Kim TA Nakano M Iida Y Barrett JC Oshimura M Masumoto H Earnshaw WC Larionov V Kouprina N Human artificial chromosome (HAC) vector with a conditional centromere for correction of genetic deficiencies in human cells Proc Natl Acad Sci USA 2011 8 20048 20053 22123967 10.1073/pnas.1114483108 PMC3250132

[B10] Kouprina N Earnshaw WC Masumoto H Larionov V A new generation of human artificial chromosomes for functional genomics and gene therapy Cell Mol Life Sci 2012 70 1135 1148 22907415 10.1007/s00018-012-1113-3 PMC3522797

[B11] Rudd MK Mays RW Schwartz S Willard HF Human artificial chromosomes with alpha satellite-based de novo centromeres show increased frequency of nondisjunction and anaphase lag Mol Cell Biol 2003 23 7689 7697 10.1128/MCB.23.21.7689-7697.2003 14560014 PMC207596

[B12] Spence JM Mills W Mann K Huxley C Farr CJ Increased missegregation and chromosome loss with decreasing chromosome size in vertebrate cells Chromosoma 2006 115 60 74 10.1007/s00412-005-0032-6 16267674

[B13] Iida Y Kim JH Kazuki Y Hoshiya H Takiguchi M Hayashi M Erliandri I Lee HS Samoshkin A Masumoto H Earnshaw WC Kouprina N Larionov V Oshimura M Human artificial chromosome with a conditional centromere for gene delivery and gene expression DNA Res 2010 17 293 301 10.1093/dnares/dsq020 20798231 PMC2955713

[B14] Koi M Shimizu M Morita H Yamada H Oshimura M Construction of mouse A9 clones containing a single human chromosome tagged with neomycin-resistance gene via microcell fusion Jpn J Cancer Res 1989 80 413 418 10.1111/j.1349-7006.1989.tb02329.x 2502516 PMC5917765

[B15] Kim JH Ebersole T Kouprina N Noskov VN Ohzeki JI Masumoto H Mravinac B Sullivan BA Dovat S Pavlicek A Pack SD Kwon YW Flanagan PT Loukinov D Lobanenkov V Larionov V Human pericentromeric gamma-satellite DNA maintains open chromatin structure and protects a transgene from epigenetic silencing at an ectopic site Genome Res 2009 19 533 544 10.1101/gr.086496.108 19141594 PMC2665773

[B16] Ebersole T Kim JH Samoshkin A Kouprina N Pavlicek A White R Larionov V tRNA genes protect a reporter gene from epigenetic silencing in mouse cells Cell Cycle 2011 10 2729 2791 10.4161/cc.10.16.17092 PMC3219543 21822054

[B17] Ikeno M Grimes B Okazaki T Nakano M Saitoh K Hoshino H McGill NI Cooke H Masumoto H Construction of YAC-based mammalian artificial chromosomes Nat Biotechnol 1998 16 431 439 9592390 10.1038/nbt0598-431

[B18] Fenech M Cytokinesis-block micronucleus cytome assay Nat Protoc 2007 2 1084 1104 10.1038/nprot.2007.77 17546000

[B19] Dumontet C Jordan MA Microtubule-binding agents: a dynamic field of cancer therapeutics Nat Rev Drug Discov 2010 9 790 803 10.1038/nrd3253 20885410 PMC3194401

[B20] Georgieva I Koychev D Wang Y Holstein J Hopfenmüller W Zeitz M Grabowski P ZM447439, a novel promising aurora kinase inhibitor, provokes antiproliferative and proapoptotic effects alone and in combination with bio- and chemotherapeutic agents in gastroenteropancreatic neuroendocrine tumor cell lines Neuroendocrinology 2010 91 121 130 10.1159/000258705 19923785

[B21] Santaguida S Tighe A D'Alise AM Taylor SS Musacchio A Dissecting the role of MPS1 in chromosome biorientation and the spindle checkpoint through the small molecule inhibitor reversine J Cell Biol 2010 190 73 87 10.1083/jcb.201001036 20624901 PMC2911657

[B22] Robbins AR Jablonski SA Yen TJ Yoda K Robey R Bates SE Sackett DL Inhibitors of histone deacetylases alter kinetochore assembly by disrupting pericentromeric heterochromatin Cell Cycle 2005 4 717 726 10.4161/cc.4.5.1690 15846093

[B23] Pommier Y Leo E Zhang H Marchand C DNA topoisomerases and their poisoning by anticancer and antibacterial drugs Chem Biol 2010 17 421 433 10.1016/j.chembiol.2010.04.012 20534341 PMC7316379

[B24] Kirkland D Pfuhler S Tweats D Aardema M Corvi R Darroudi F Elhajouji A Glatt H Hastwell P Hayashi M Kasper P Kirchner S Lynch A Marzin D Maurici D Meunier JR Müller L Nohynek G Parry J Parry E Thybaud V Tice R Van Benthem J Vanparys P White P How to reduce false positive results when undertaking in vitro genotoxicity testing and thus avoid unnecessary follow-up animal tests: Report of an ECVAM Workshop Mutat Res 2007 628 31 55 10.1016/j.mrgentox.2006.11.008 17293159

[B25] Hashimoto K Nakajima Y Matsumura S Chatani F An in vitro micronucleus assay with size-classified micronucleus counting to discriminate aneugens from clastogens Toxicol In Vitro 2010 24 208 216 10.1016/j.tiv.2009.09.006 19747535

[B26] Ingemarsdotter CK Baird SK Connell CM Öberg D Halldén G McNeish IA Low-dose paclitaxel synergizes with oncolytic adenoviruses via mitotic slippage and apoptosis in ovarian cancer Oncogene 2010 29 6051 6063 10.1038/onc.2010.335 20729921 PMC3007619

[B27] Elhajouji A Lukamowicz M Cammerer Z Kirsch-Volders M Potential thresholds for genotoxic effects by micronucleus scoring Mutagenesis 2011 26 199 204 10.1093/mutage/geq089 21164203

[B28] Thompson SL Compton DA Examining the link between chromosome instability and aneuploidy in human cells J Cell Biol 2008 180 665 672 10.1083/jcb.200712029 18283116 PMC2265570

[B29] Asur RS Thomas RA Tucker JD Chemical induction of the bystander effect in normal human lymphoblastoid cells Mutat Res 2009 676 11 16 10.1016/j.mrgentox.2009.02.012 19486859

[B30] Meintières S Marzin D Apoptosis may contribute to false-positive results in the in vitro micronucleus test performed in extreme osmolality, ionic strength and pH conditions Mutat Res 2004 560 101 118 10.1016/j.mrgentox.2004.02.003 15157649

[B31] Crasta K Ganem NJ Dagher R Lantermann AB Ivanova EV Pan Y Nezi L Protopopov A Chowdhury D Pellman D DNA breaks and chromosome pulverization from errors in mitosis Nature 2012 482 53 58 10.1038/nature10802 22258507 PMC3271137

[B32] Burns EM Christopoulou L Corish P Tyler-Smith C Quantitative measurement of mammalian chromosome mitotic loss rates using the green fluorescent protein J Cell Sci 1999 112 2705 2714 10413678 10.1242/jcs.112.16.2705

[B33] Ohzeki JI Bergmann JH Kouprina N Noskov VN Nakano M Kimura H Earnshaw WC Larionov V Masumoto H Breaking the HAC Barrier: Histone H3K9 acetyl/methyl balance regulates CENP-A assembly EMBO J 2012 31 2391 2402 10.1038/emboj.2012.82 22473132 PMC3364751

[B34] Thompson SL Compton DA Chromosome missegregation in human cells arises through specific types of kinetochore-microtubule attachment errors Proc Natl Acad Sci USA 2011 108 17974 17978 10.1073/pnas.1109720108 21997207 PMC3207692

[B35] Spencer F Gerring SL Connelly C Hieter P Mitotic chromosome transmission fidelity mutants in *Saccharomyces cerevisiae* Genetics 1990 124 237 249 2407610 10.1093/genetics/124.2.237 PMC1203917

