# Supplementary material for: A new assay for measuring chromosome instability (CIN) and identification of drugs that elevate CIN in cancer cells
Source: BMC Cancer. 2013 May 22;13:252. doi: 10.1186/1471-2407-13-252 (PMC3671967; doi:10.1186/1471-2407-13-252)
Supplement: Additional file 1: Figure S1. — (A) Micronuclei (MNi) formation (indicated by arrows) in the HAC-containing HT1080 cells treated by drugs. Scale bar = 10 μM. (B) The kinetics of MNi formation after drug treatment at different time points after washout. The experiment was performed for taxol (10 nM) and nocodazole (1 μM). (C) Rates of HAC loss calculated using FACS profiles of cells before, after drug treatment and at different time points after washout. The experiment was performed for taxol and ixabepilone. [file 1471-2407-13-252-S1.doc]

Additional File 1


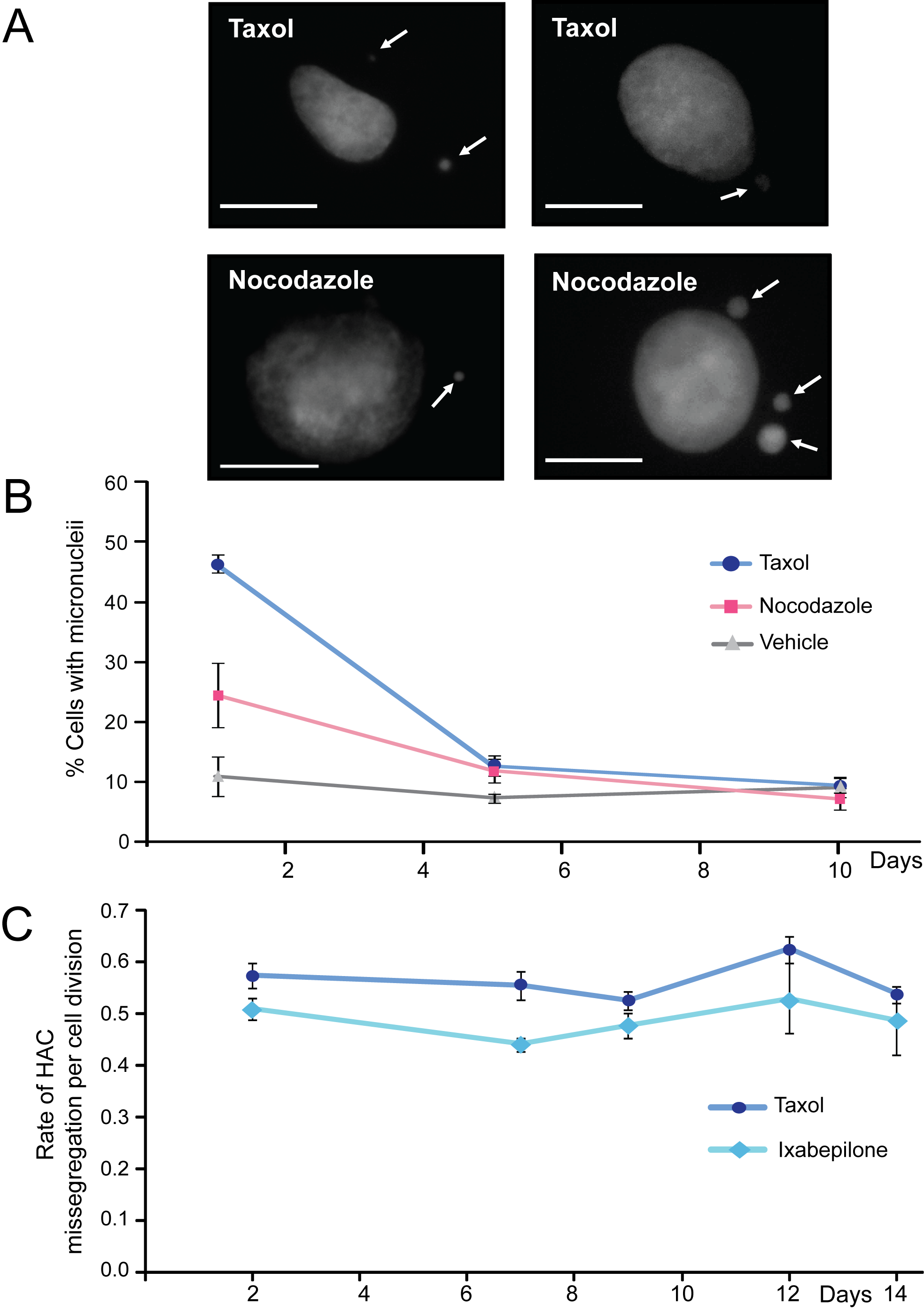


**Figure S1** **(A)** Micronuclei (MNi) formation (indicated by arrows) in the HAC-containing HT1080 cells treated by drugs. Scale bar = 10 µM. **(B)** The kinetics of MNi formation after drug treatment at different time points after washout. The experiment was performed for taxol (10 nM) and nocodazole (1 µM). **(C)** Rates of HAC loss calculated using FACS profiles of cells before, after drug treatment and at different time points after washout. The experiment was performed for taxol and ixabepilone .
